# A Randomized Clinical Trial Comparing Immediate Loading and Delayed Loading of Single-Tooth Implants: 5-Year Results

**DOI:** 10.3390/jcm10051077

**Published:** 2021-03-05

**Authors:** Björn Gjelvold, Jenö Kisch, Bruno R. Chrcanovic

**Affiliations:** 1 Clinic for Prosthodontics, Centre of Dental Specialist Care, 214 27 Malmö, Sweden; bjorn.gjelvold@gmail.com (B.G.); jeno.kisch@mau.se (J.K.); 2 Department of Prosthodontics, Faculty of Odontology, Malmö University, 214 21 Malmö, Sweden

**Keywords:** dental implant, immediate loading, delayed loading, survival, implant-supported crown, patient satisfaction, randomized controlled trial

## Abstract

The aim of this prospective randomized clinical study was to compare the clinical treatment outcome for single dental implants submitted to either immediate loading (IL) or delayed loading (DL) after 5 years of follow-up. Fifty patients with a missing maxillary tooth (15–25) were randomly allocated to either the IL or DL. The treatment procedures included implant installation in healed sites, temporary screw-retained crown and replacement with a permanent single implant crown. The two groups were evaluated with regard to implant survival, marginal bone level, papillae index, pink and white esthetic score (PES, WES). At the 5-year follow-up the implant survival rate was 100% and 95.8% for IL and DL, respectively. Implant success rate was 91.7% and 83.3% for IL and DL, respectively. The mean ± SD marginal bone loss for IL and DL was −0.50 ± 0.73 mm and −0.54 ± 0.65 mm, respectively. (*p* = 0.782). Statistically significant less marginal bone loss was found non-smokers (*p* = 0.021). No statistically significant differences were found for IL and DL concerning papillae index PES and WES after 5 years. This study suggests that implant-supported single crowns in the maxillary aesthetic zone can present similar results with respect to either IL or DL after 5 years.

## 1. Introduction

Single crowns supported by implants have become a well-established approach to rehabilitate single-tooth edentulous spaces, especially when the adjacent teeth are in pristine state [1]. When the rehabilitation site is in the anterior maxilla, the aesthetic appearance is of paramount importance [2]. This is especially true for when the patient requests immediate function of the implant-supported crown over delayed loading (DL), which could have aesthetical short-term implications [3]. A previous study observed lower papilla index scores for the immediate loading (IL) single crowns at temporary and definitive crown placement stages in comparison to DL crowns [3].

The present 5-year randomized clinical study aimed to compare clinical and aesthetic outcomes of implant-supported single crowns in the aesthetic zone of the maxilla submitted to either IL and DL protocols. This report consists of longer clinical follow-up results of a previously published 1-year clinical study [3].

## 2. Materials and Methods

The present prospective randomized clinical trial was conducted in compliance with the Helsinki declaration [4]. The study protocol was approved by the Regional Ethical Review Board in Lund, Sweden (Dnr 2011/125) and was registered in ClinicalTrails.gov (ID: NCT02770846).

Patients referred to the Centre of Dental Specialist Care, Malmö between April 2011 and April 2014 were considered for inclusion in the present study. In order to be able to be included, patients needed (1) to be 18 years old or older, (2) to have a single-tooth edentulous space between maxillary teeth 15–25, (3) to have the implant site with healed bone, i.e., at least 4 months after tooth extraction, or tooth agenesis, and (4) to have agreed to take part in the study have signed an informed consent.

Exclusion criteria consisted of (1) patients with general health contraindications for oral surgery, (2) inadequate oral hygiene, defined as a full-mouth plaque score of above 25%, and (3) patients in need of bone grafting or ridge augmentation at the implant site.

**Figure 1:**
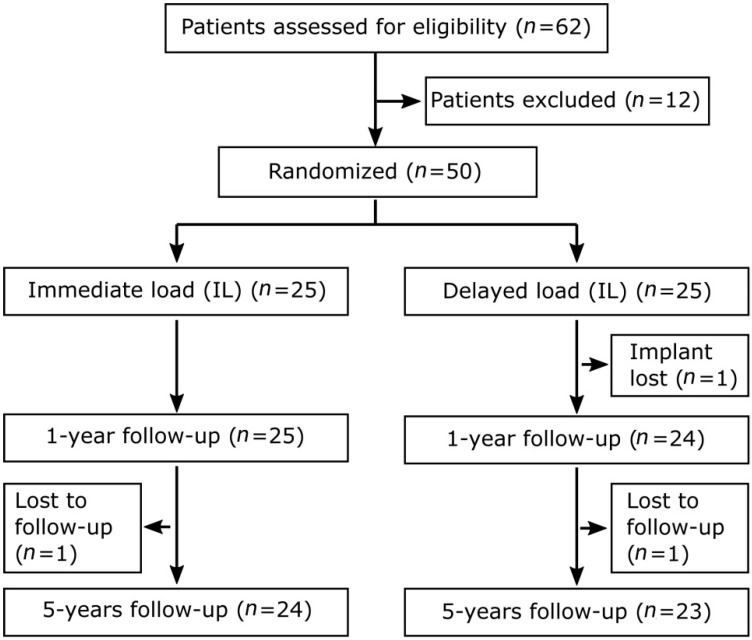
Clinical trial outline of study participants.

A number of 50 patients distributed in two groups was determined as acceptable to reach the level of required statistical power (80% power, with a margin of error of 5%, and confidence level of 95%). Sixty-two patients were initially recruited, of which twelve patients were excluded for reasons that have been previously described in detail [3]. The remaining 50 patients were allocated to either the IL or DL group by randomization. For the clinical trial outline, see Figure 1.

The patients were randomized using a sealed envelope lot. The surgeon (J.K.) who placed the implants was blinded regarding the assignment to different treatment groups. All patients received Tapered Internal implants (BioHorizons, Birmingham, AL, USA). The implant placement was conducted as per manufacturer recommendations, namely at least half the Laser-Lok collar surface of the fixture’s neck had contact with bone tissue. None of the implants were placed in a subcrestal position. All implants in the IL group reached the minimum insertion torque value of 30 Ncm. One implant from the DL group failed 3 months after surgical placement. Details of gender, smoker/non-smoker, presurgical evaluation, surgical techniques and restorative procedures have been previously described in detail [3]. For implant locations, see Table 1.

In short, implants in the IL group were immediately loaded with a screw-retained temporary crown adjusted to a light centric contact and free from eccentric contacts during two months, after which restored with the definitive restoration. In the DL group a two-stage surgery procedure was adopted, and there was a 4-month postoperative healing before a screw-retained temporary crown was used to shape the emergence profile prior to delivery of the final restoration. All definitive crowns consisted of a titanium base (Medentica GmbH, Hügelsheim, Germany), an individually fabricated zirconia abutment (I-butment, Biomain AB, Helsingborg, Sweden) and were veneered (GC Initial, GC EUROPE N.V., Leuven, Belgium) by the same dental technician. Screw-retained crowns were the aim for all cases. When the screw-retained approach was not possible due to aesthetic reasons the crown was cemented. All cases were thoroughly checked for excess cement with periapical radiographs, visual inspection, and probing.

### 2.1. Follow-Up Appointments and Evaluations

Clinical and radiographic follow-up examination were conducted at 3, 6, 12 months and 5 years after definitive crown placement. Details regarding the 3, 6 and 12 months results have been previously described in detail [3]. The implant surgery day was considered as baseline for the radiographic follow-up, and placement of the definitive crown for the aesthetic evaluations. All clinical examinations were carried out by the same examiner (B.G.), who was responsible for the prosthetic treatment. The assessment of marginal bone loss (MBL) and aesthetics was performed by an examiner (B.R.C.) not involved in patient treatment and blinded to patient group allocation.

All patients attended the 12-month follow-up. At the 5-year follow-up, the total number of unaccounted patients was two (4.0%): one patient in the DL group was not possible to contact and one patient in the IL group did not want to attend the clinical consultation. The later patient confirmed that the implant and restoration were still in place and that he had not experienced any problems, which was considered as survival.

The evaluation of implant success and survival was performed according to some previous guidelines [5,6]. Implants not lost were considered as survival regardless of condition. Success, a criterion for MBL over time, was defined as a maximum 1.0 mm of MBL during the first year and <0.2 mm annually thereafter, besides absence of implant mobility and/or pain, peri-implant radiolucency, and infection.

**Figure 2:**
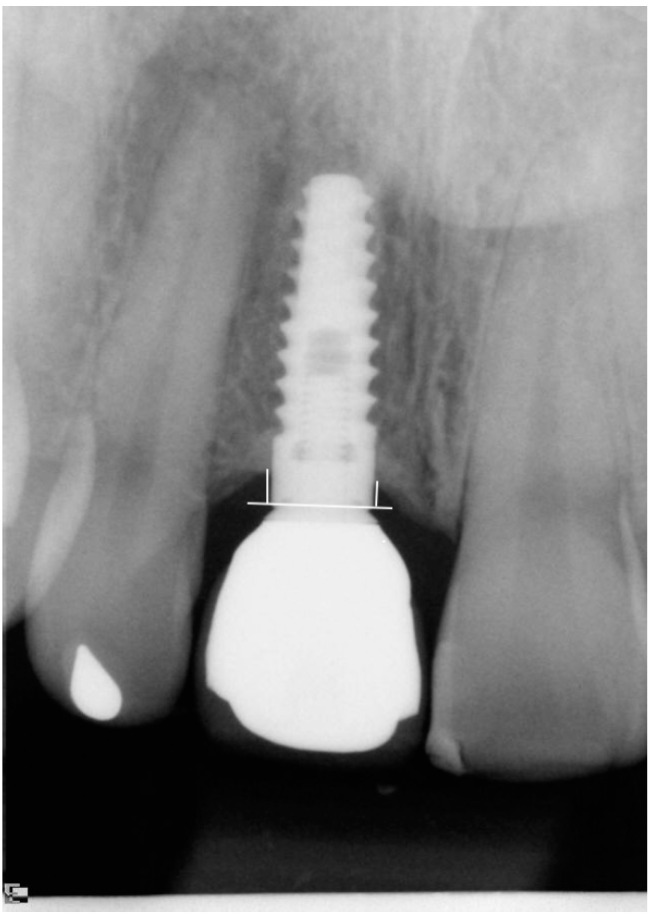
Marginal bone level measurement. A reference line is drawn at the implant-abutment junction from where the mesial and distal marginal bone levels are measured.

For the assessment of MBL digital intra-oral periapical radiographs (Schick Digital X-ray Sensor, Sirona, Salzburg, Austria) were taken using the long-cone parallel technique. The marginal bone level was measured (Image J, National Institute of Health, Bethesda, MD, USA) after calibration with the inter-thread distance of the Tapered Internal implants (1.00 mm). Measurements were taken from the implant-abutment junction to the marginal bone level. The mean value from the mesial and distal measurements was obtained and used for the analysis (Figure 2). MBL was calculated by comparing the follow-up examinations to the baseline examination.

The papilla index [7] was scored for the mesial and distal papilla at baseline and at each follow-up examination.

Intraoral photographs from baseline and the follow-up examinations were used to register the pink esthetic score (PES) [8] and the white esthetic score (WES) [9]. The scores were defined according to a previous publication, as (almost) perfect outcome for PES and WES as PES ≥ 12 and WES ≥ 9, respectively, aesthetic failure as PES ≤ 7 or WES ≤ 5, respectively, and as complete aesthetic failure as PES ≤ 7 and WES ≤ 5, respectively [10].

### 2.2. Statistics

All data were statistically analyzed by one examiner, who did not take part in any of the clinical procedures. The software used was the Statistical Package for the Social Sciences (SPSS) version 26 (SPSS Inc., Chicago, IL, USA). Mean, standard deviation (SD), minimum and maximum values were calculated for several variables. For all calculations, missing values were excluded list-wise for the 1-year and 5-year follow-up. Normality was checked by Kolmogorov–Smirnov test, and homoscedasticity was evaluated by the Levene’s test. The performed tests for two independent groups and two dependent groups were Student’s *t*-test or Mann–Whitney test, depending on the normality. Pearson’s chi-squared or Fisher’s exact test was performed for categorical variables. The degree of statistical significance was considered *p* < 0.05.

## 3. Results

The 5-year implant survival rate was 100% and 95.8% for IL and DL, respectively. The 5-year implant success rate was 91.7% and 83.3% for IL and DL groups, respectively.

The occurrence of redness and edema in combination with bleeding on probing at the 5-year follow-up was 8.5% for both groups, and 4.3% and 12.5% for DL and IL groups, respectively. A technical complication occurred in one single crown of the DL group (4.3%), loss of retention of the titanium base (52 months after baseline). Further, one crown (4.2%) in the IL group was replaced due to aesthetic reasons after veneering of adjacent teeth (47 months after baseline), and this was not considered as a technical complication.

### 3.1. Marginal Bone Loss and Papilla Index

The 1-year mean ± SD (min, max) MBL for IL and DL was −0.57 ± 0.53 mm (0.21, −2.05) and −0.70 ± 0.58 mm (0.18, −2.37), respectively (*p* = 0.430, Student’s *t*-test). At the 5-year follow-up the mean ± SD (min, max) MBL for IL and DL was −0.50 ± 0.73 mm (0.45, −2.81) and −0.54 ± 0.65 mm (0.36, −2.37), respectively (*p* = 0.782, Mann–Whitney test). For the comparisons of MBL between different subgroups, see Table 2. Statistical significant difference was found for the overall MBL between smokers and non-smokers (*p* = 0.021, Mann–Whitney test).

The results for papilla index are presented in Table 3. Patients with a complete papilla fill on both mesial and distal sides in IL and DL at the 5-year follow-up were 33.3% and 56.5%, respectively (*p* = 0.147, Fisher’s exact test). In comparison, the 1-year results for IL and DL were 29.2% and 47.8%, respectively (*p* = 0.238, Fisher’s exact test).

### 3.2. Aesthetic Scores

The results for PES and WES concerning IL and DL are presented in Table 4. There were no statistically significant differences between the two groups. There was a statistically significant improvement in PES between the 1-year and 5-year follow-up for both IL and DL (*p* = 0.017 and *p* = 0.046, Wilcoxon signed-rank test), contrary to WES (*p* = 0.564 and *p* = 1.000, Wilcoxon signed-rank test).

The overall aesthetic outcome after 1 year was assessed by combining PES and WES. Almost perfect outcome (PES ≥ 12 and WES ≥ 9) for IL and DL were found for 20.8% (*n* = 5) and 17.4% (*n* = 4) of the crowns, respectively. Acceptable results were found for 62.5% (*n* = 15) and 69.6% (*n* = 16) of the IL and DL cases, respectively. Aesthetic failure (PES ≤ 7 and/or WES ≤ 5) were found for 16.7% (*n* = 4) and 13.0% (*n* = 3) of the IL and DL cases, respectively. Complete aesthetic failure (PES ≤ 7 and WES ≤ 5) were found for 4.2% (*n* = 1) and 8.7% (*n* = 2) of the IL and DL cases, respectively.

The overall aesthetic outcome after 5 years was almost perfect outcome (PES ≥ 12 and WES ≥ 9) for 20.8% (*n* = 5) and 30.4% (*n* = 7) of the IL and DL cases, respectively. Acceptable results were found for 66.7% (*n* = 16) and 56.5% (*n* = 13) of the IL and DL cases, respectively. Aesthetic failure (PES ≤ 7 or WES ≤ 5) was found for 12.5% (*n* = 3) and 13.0% (*n* = 3) of the IL and DL cases, respectively. Complete aesthetic failure (PES ≤ 7 and WES ≤ 5) were found for 4.2% (*n* = 1) and 8.7% (*n* = 2) of the IL and DL cases, respectively.

**Figure 3:**
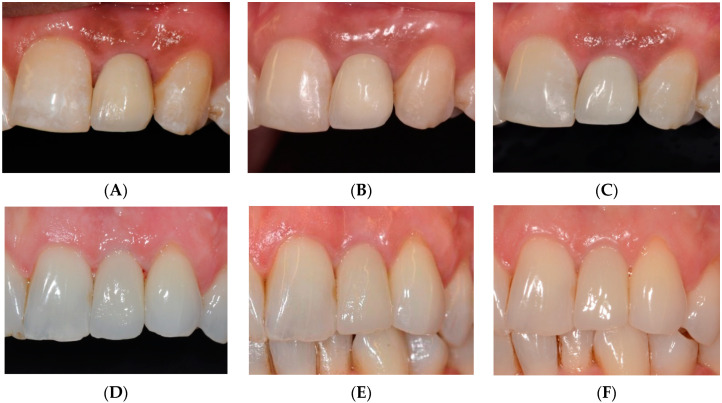
Examples of photograph follow-up for both groups. (**A**) Delivery of final restoration IL, (**B**) 1-year follow-up IL, (**C**) 5-year follow-up IL, (**D**) Delivery of final restoration DL, (**E**) 1-year follow-up DL, (**F**) 5-year follow-up DL.

Figure 3 shows examples of photograph follow-up of the present study for both groups (IL and DL, respectively).

## 4. Discussion

The present 5-year study aimed to compare the clinical and aesthetic outcomes of implant-supported single crowns in the aesthetic zone of the maxilla submitted to either IL or DL protocols, providing longer follow-up results of a previously published paper that reported short-term 1-year results [3]. We considered that the rate of patients unaccounted for was considerably low (4%) after 5 years. Additionally, this is important, since poor recruitment is a crucial factor in conducting randomized clinical trials, as inadequate recruitment may cause a level of uncertainty about the treatment efficacy [11].

The 5-year implant survival rate was 100% and 95.8% for IL and DL, respectively, the same as presented at the 1-year follow-up. The only lost implant failed 3 months after surgery, in a patient who was a smoker, a habit that could have contributed for this early negative result [12,13].

Although many studies have shown high survival rates, implant-supported dental prostheses still present complications, and their longevity is influenced by biologic and technical complications, as well as by prosthetic maintenance requirements [14,15,16,17]. Only two complications occurred during the entire follow-up of the study, a loss of retention of the titanium base, a technical complication, and the replacement of a crown due to aesthetic reasons, a non-technical complication. This is in agreement with a recent clinical study that evaluated implant-supported single crown for a mean follow-up of 15 years, that observed that the occurrence of non-technical complications are as common as technical ones in implant-supported single crowns [1].

The mean marginal bone resorption around the implants remained quite similar between the two groups after 5 years, with even a small mean bone gain between the 1- and 5-year follow-up appointments. As in the 1-year follow-up, there was a lack of statistically significant difference for MBL between the two groups, showing that the difference in the loading protocol did not affect the MBL after a longer observation time, in agreement with other studies [18,19].

The 1-year follow-up study [3] showed a statistically significant difference in papilla index between the two groups. The quantitative difference in the results of the index between the groups became smaller with time, also tending to show no clear clinical difference in the long term, adding evidence that a longer follow-up can result in additional papilla formation [7]. The progressive increase in aesthetics and changes in soft tissue shape are related to the gradual papillae formation and the healing process of the mucosa with time [20]. In that sense, it is anticipated that both PES and WES will get higher scores with time, as the perception of the crown shape and contour are highly influenced by the improvement of the soft tissue around the single crowns [3]. The increase in PES and WES mean values were also reflected in the percentage of cases categorized as aesthetic failure and almost perfect outcome, a slight increase in cases with almost perfect outcome in the DL-group and a decrease in cases with aesthetic failures for the IL-group. The present rate of perfect outcome and aesthetic failure falls within the range reported by others [9,10,21,22]. Complete failures related to both PES and WES were in minority and did not change over time. The good PES-WES scores could be also a result of the low MBL after 5 years, as the small amount of peri-implant MBL did not result a clinical effect that was enough to be observed with the indexes, as also shown in another study [23]. The fact that only single implants with natural roots on both sides were included is another possible factor that could have helped in obtaining good aesthetic scores, as it is recognized that the marginal bone level in vicinity to the adjacent teeth is related to the level of the papillae [24], and none of the teeth being replaced by an implant in this study was lost for periodontal reasons [3].

## 5. Conclusions

The present prospective randomized study observed that implant-supported single crowns in the maxillary aesthetic zone can present similar aesthetic and clinical results after 5 years of follow-up, regardless of whether submitted to either immediate loading or delayed loading.

## Figures and Tables

**Table 1:** Overview of implants according to their location.

Location, FDI *	**15**	**14**	**13**	**12**	**11**
Immediate loading	4	2	0	2	3
Implant *n* (%)	(16.0)	(8.0)	(0.0)	(8.0)	(12.0)
Delayed loading	3	2	1	1	3
Implant *n* (%)	(12.0)	(8.0)	(4.0)	(4.0)	(12.0)
Location, FDI *	21	22	23	24	25
Immediate loading	2	3	3	3	3
Implant *n* (%)	(8.0)	(12.0)	(12.0)	(12.0)	(12.0)
Delayed loading	6	2	3	3	1
Implant *n* (%)	(24.0)	(8.0)	(12.0)	(12.0)	(4.0)

* Fédération Dentaire Internationale (FDI) notation system, ISO 3950.

**Table 2:** Comparison of MBL between different groups after 5 years.

	Mean ± SD (Min, Max)	*n*	Mean ± SD (Min, Max)	*n*	*p* Value *
**Tobacco**	**Smokers**		**Nonsmokers**		
Immediate loading	−1.86 ± 1.33 (−0.93, −2.81)	2	−0.37 ± 0.55 (0.45, −1.79)	22	0.060
Delayed loading	−0.91 ± 0.66 (−0.23, −1.63)	5	−0.44 ± 0.62 (0.36, −2.37)	18	0.180
Total	−1.90 ± 0.90 (−0.23, −2.81)	7	−0.40 ± 0.58 (0.45, −2.37)	40	0.021
**Sex**	**Male**		**Female**		
Immediate loading	−0.68 ± 0.85 (0.44, −2.81)	13	−0.28 ± 0.51 (0.45, −1.09)	11	0.213
Delayed loading	−0.44 ± 0.33 (0.11, −0.73)	6	−0.57 ± 0.73 (0.36, −2.37)	17	1.000
Total	−0.61 ± 0.72 (0.44, −2.81)	19	−0.46 ± 0.66 (0.45, −2.37)	28	0.374
**Cemented/screw-retained**	**Cemented**		**Screw-retained**		
Immediate loading	−0.46 ± 0.70 (0.45, −1,79)	9	−0.52 ± 0.77 (0.44, −2.81)	15	0.976
Delayed loading	−0.55 ± 0.84 (0.36, −2.37)	8	−0.53 ± 0.55 (0.34, −1.63)	15	0.796
Total	−0.51 ± 0.74 (0.45, −2.37)	17	−0.52 ± 0.66 (0.44, −2.81)	30	0.808

SD—standard deviation; MBL—marginal bone loss in mm (negative values represent bone loss); * Mann–Whitney test.

**Table 3:** Papilla index results.

	Mean ± SD (Min, Max)	*n*	Mean ± SD (Min, Max)	*n*	*p* Value *
**Papilla index, mesial**	**Immediate loading**		**Delayed loading**		
Definitive crown placement	1.88 ± 0.99 (0, 3)	24	2.26 ± 0.81 (0, 3)	23	0.169
1-year follow-up	2.54 ± 0.51 (2, 3)	24	2.61 ± 0.58 (1, 3)	23	0.534
5 years follow-up	2.54 ± 0.51 (2, 3)	24	2.57 ± 0.73 (1, 3)	23	0.518
**Papilla index, distal**					
Definitive crown placement	1.21 ± 0.88 (0, 3)	24	2.09 ± 0.85 (0, 3)	23	0.002
1-year follow-up	2.13 ± 0.68 (1, 3)	24	2.26 ± 0.86 (0, 3)	23	0.362
5 years follow-up	2.33 ± 0.67 (1, 3)	24	2.39 ± 0.84 (1, 3)	23	0.527

SD—standard deviation; MBL—marginal bone loss (negative values represent bone loss); * Mann–Whitney test.

**Table 4:** Aesthetic score results.

	Mean ± SD (Min, Max)	*n*	Mean ± SD (Min, Max)	*n*	*p* Value
**PES**	**Immediate loading**		**Delayed loading**		
Definitive crown placement	8.58 ± 2.32 (2, 13)	24	9.48 ± 3.03 (4, 14)	23	0.260 ^†^
1-year follow-up	10.38 ± 2.43 (3, 14)	24	10.83 ± 2.42 (5, 14)	23	0.517 *
5 years follow-up	11.00 ± 2.08 (5, 14)	24	11.17 ± 2.39 (5, 14)	23	0.501 *
**WES**					
Definitive crown placement	7.00 ± 1.44 (4, 10)	24	7.09 ± 1.62 (4, 10)	23	0.983 *
1-year follow-up	7.71 ± 1.30 (5, 10)	24	7.91 ± 1.41 (5, 10)	23	0.521 *
5 years follow-up	7.63 ± 1.53 (5, 10)	24	7.91 ± 1.53 (4, 10)	23	0.365 *

SD—standard deviation; ^†^ Student’s *t*-test; * Mann–Whitney test.

## Data Availability

Restrictions apply to the availability of these data. Data was obtained from patients treated at Folktandvården Skåne AB, Malmö, Sweden, and cannot be shared, in accordance with the General Data Protection Regulation (EU) 2016/679.

## References

[1] Chrcanovic B.R., Kisch J., Larsson C. (2019). Retrospective clinical evaluation of implant-supported single crowns: Mean follow-up of 15 years. Clin. Oral Implant. Res..

[2] Gjelvold B., Chrcanovic B.R., Bagewitz I.C., Kisch J., Albrektsson T., Wennerberg A. (2017). Esthetic and Patient-Centered Outcomes of Single Implants: A Retrospective Study. Int. J. Oral Maxillofac. Implant..

[3] Gjelvold B., Kisch J., Chrcanovic B.R., Albrektsson T., Wennerberg A. (2017). Clinical and radiographic outcome following immediate loading and delayed loading of single-tooth implants: Randomized clinical trial. Clin. Implant. Dent. Relat. Res..

[4] World Medical Association (2000). Revising the Declaration of Helsinki. Bull. Med. Ethics..

[5] Albrektsson T., Zarb G., Worthington P., Eriksson A.R. (1986). The long-term efficacy of currently used dental implants: A review and proposed criteria of success. Int. J. Oral Maxillofac. Implant..

[6] Albrektsson T., Zarb G.A. (1993). Current interpretations of the osseointegrated response: Clinical significance. Int. J. Prosthodont..

[7] Jemt T. (1997). Regeneration of gingival papillae after single-implant treatment. Int. J. Periodontics Restor. Dent..

[8] Fürhauser R., Florescu D., Benesch T., Haas R., Mailath G., Watzek G. (2005). Evaluation of soft tissue around single-tooth implant crowns: The pink esthetic score. Clin. Oral Implant. Res..

[9] Belser U.C., Grütter L., Vailati F., Bornstein M.M., Weber H.P., Buser D. (2009). Outcome evaluation of early placed maxillary anterior single-tooth implants using objective esthetic criteria: A cross-sectional, retrospective study in 45 patients with a 2- to 4-year follow-up using pink and white esthetic scores. J. Periodontol..

[10] Cosyn J., Eghbali A., De Bruyn H., Dierens M., De Rouck T. (2012). Single implant treatment in healing versus healed sites of the anterior maxilla: An aesthetic evaluation. Clin. Implant. Dent. Relat. Res.

[11] Kadam R.A., Borde S.U., Madas S.A., Salvi S.S., Limaye S.S. (2016). Challenges in recruitment and retention of clinical trial subjects. Perspect. Clin. Res..

[12] Chrcanovic B.R., Albrektsson T., Wennerberg A. (2015). Smoking and dental implants: A systematic review and meta-analysis. J. Dent..

[13] Chrcanovic B.R., Kisch J., Albrektsson T., Wennerberg A. (2016). Factors Influencing Early Dental Implant Failures. J. Dent. Res..

[14] Chrcanovic B.R., Ghiasi P., Kisch J., Lindh L., Larsson C. (2020). Retrospective study comparing the clinical outcomes of bar-clip and ball attachment implant-supported overdentures. J. Oral Sci..

[15] Chrcanovic B.R., Kisch J., Larsson C. (2020). Retrospective clinical evaluation of 2- to 6-unit implant-supported fixed partial dentures: Mean follow-up of 9 years. Clin. Implant. Dent. Relat. Res..

[16] Chrcanovic B.R., Kisch J., Larsson C. (2020). Retrospective evaluation of implant-supported full-arch fixed dental prostheses after a mean follow-up of 10 years. Clin. Oral Implant. Res..

[17] Chrcanovic B.R., Kisch J., Larsson C. (2020). Analysis of technical complications and risk factors for failure of combined tooth-implant-supported fixed dental prostheses. Clin. Implant. Dent. Relat. Res..

[18] Chidagam P., Gande V.C., Yadlapalli S., Venkata R.Y., Kondaka S., Chedalawada S. (2017). Immediate Versus Delayed Loading of Implant for Replacement of Missing Mandibular First Molar: A Randomized Prospective Six Years Clinical Study. J. Clin. Diagn. Res..

[19] Meloni S.M., Baldoni E., Duvina M., Pisano M., De Riu G., Tallarico M. (2018). Immediate non-occlusal versus delayed loading of mandibular first molars. Five-year results from a randomised controlled trial. Eur. J. Oral Implantol..

[20] Sculean A., Gruber R., Bosshardt D.D. (2014). Soft tissue wound healing around teeth and dental implants. J. Clin. Periodontol..

[21] Buser D., Halbritter S., Hart C., Bornstein M.M., Grütter L., Chappuis V., Belser U.C. (2009). Early implant placement with simultaneous guided bone regeneration following single-tooth extraction in the esthetic zone: 12-month results of a prospective study with 20 consecutive patients. J. Periodontol..

[22] Meijndert L., Meijer H.J., Stellingsma K., Stegenga B., Raghoebar G.M. (2007). Evaluation of aesthetics of implant-supported single-tooth replacements using different bone augmentation procedures: A prospective randomized clinical study. Clin. Oral Implant. Res..

[23] Den Hartog L., Raghoebar G.M., Slater J.J., Stellingsma K., Vissink A., Meijer H.J. (2013). Single-tooth implants with different neck designs: A randomized clinical trial evaluating the aesthetic outcome. Clin. Implant. Dent. Relat. Res..

[24] Choquet V., Hermans M., Adriaenssens P., Daelemans P., Tarnow D.P., Malevez C. (2001). Clinical and radiographic evaluation of the papilla level adjacent to single-tooth dental implants. A retrospective study in the maxillary anterior region. J. Periodontol..

